# Long-wavelength sensitive visual pigments of the guppy (*Poecilia reticulata*): six opsins expressed in a single individual

**DOI:** 10.1186/1471-2148-7-S1-S11

**Published:** 2007-02-08

**Authors:** Cameron J Weadick, Belinda SW Chang

**Affiliations:** 1 Departments of Ecology & Evolutionary Biology, Cell & Systems Biology, and Centre for the Analysis of Genome Evolution & Function, University of Toronto, 25 Harbord Street, M5S3G5, Ontario, Canada

## Abstract

**Background:**

The diversity of visual systems in fish has long been of interest for evolutionary biologists and neurophysiologists, and has recently begun to attract the attention of molecular evolutionary geneticists. Several recent studies on the copy number and genomic organization of visual pigment proteins, the opsins, have revealed an increased opsin diversity in fish relative to most vertebrates, brought about through recent instances of opsin duplication and divergence. However, for the subfamily of opsin genes that mediate vision at the long-wavelength end of the spectrum, the LWS opsins, it appears that most fishes possess only one or two loci, a value comparable to most other vertebrates. Here, we characterize the LWS opsins from cDNA of an individual guppy, *Poecilia reticulata*, a fish that is known exhibit variation in its long-wavelength sensitive visual system, mate preferences and colour patterns.

**Results:**

We identified six LWS opsins expressed within a single individual. Phylogenetic analysis revealed that these opsins descend from duplication events both pre-dating and following the divergence of the guppy lineage from that of the bluefin killifish, *Lucania goodei*, the closest species for which comparable data exists. Numerous amino acid substitutions exist among these different LWS opsins, many at sites known to be important for visual pigment function, including spectral sensitivity and G-protein activation. Likelihood analyses using codon-based models of evolution reveal significant changes in selective constraint along two of the guppy LWS opsin lineages.

**Conclusion:**

The guppy displays an unusually high number of LWS opsins compared to other fish, and to vertebrates in general. Observing both substitutions at functionally important sites and the persistence of lineages across species boundaries suggests that these opsins might have functionally different roles, especially with regard to G-protein activation. The reasons why are currently unknown, but may relate to aspects of the guppy's behavioural ecology, in which both male colour patterns and the female mate preferences for these colour patterns experience strong, highly variable selection pressures.

## Background

An organism's ability to perceive, and respond to the challenges arising in its environment depends critically on the specific nature and design of its sensory system. Alterations in the molecular components of the transduction cascade can have profound consequences for sensory perception, offering the opportunity to better understand the evolution of these molecular pathways in the context of the environments in which an animal may reside. Sensory systems are therefore ideally suited for evolutionary investigations at the molecular level. For example, it has been shown that fish living at increasing depths have adapted to the changing light environment by blue-shifting their visual sensitivities via specific amino acid substitutions in one of the visual pigments that form the first step in the sensory visual pathway [1].

Visual systems are particularly amenable to molecular evolutionary analysis not only because of the diversity of light environments and visually-mediated tasks in which vision may be required, but also because the details of the primary phototransduction cascade have been fairly well characterized. In vertebrates, the first step in the visual cascade are the visual pigments, membrane-spanning protein complexes located in the outer segments of the rod and cone photoreceptor cells of the retina [2]. They are composed of two parts, an opsin protein component, which is a member of the G-protein coupled receptor superfamily, and a vitamin A-derived chromophore that is covalently bound to the opsin via a Schiff-base linkage. Absorbing a photon of light causes the chromophore to isomerize, triggering a conformational change in the opsin protein that allows it to activate the second messenger transducin, a heterotrimeric G-protein, which ultimately leads to a neural signal that light has been perceived [3,4]. Different visual pigments can be most sensitive to photons of differing wavelengths. Spectral tuning, the process by which a visual pigment's spectral sensitivity is modified, is usually achieved through amino acid substitutions that alter the environment of the chromophore within the opsin binding pocket [5,6]. Visual pigments in vertebrates are composed of five families of opsin genes [7]: the SWS1, SWS2, RH2, and LWS cone opsins that function under bright-light, or photopic conditions, and the RH1 rod opsins, or rhodopsins, that allow for vision under dim-light, or scotopic, conditions.

This dichotomy in the vertebrate visual system is reflected in the fact that cone and rod photoreceptors generally have distinct morphologies and physiological functions related to their divergent roles in day vs. night vision, with cones tending to be less photosensitive but having faster response kinetics than rods [8]. At least some of these differences are thought to be mediated by differing components of the visual transduction pathways contained within each cell type. In addition to cone visual pigments, cone-specific transducins [9], cGMP phosphodiesterases [10], cGMP-gated ion channels [11], and arrestins [12] have been found. While many of the specifics of the molecular influences on photoreceptor response kinetics remain unknown, it seems likely that some of the differences in response are due to differences in G-protein activation, as cone visual pigments are known to be less efficient in activating transducin relative to rod pigments [13].

The study of vision in fish has long attracted the attention of comparative and evolutionary biologists due to the varied and often extreme lighting conditions present in the underwater environment [14,15]. In recent years, it has been discovered that many fishes have experienced several additional rounds of opsin duplication and divergence relative to other vertebrate groups. For example, where the typical vertebrate might possess one member of each of the LWS, SWS1, SWS2, and RH2 cone opsin families, the zebrafish (*Danio rerio*) possesses two LWS, one SWS1, one SWS2, and four RH2 opsins [16], while the distantly related medaka (*Oryzias latipes*) possesses two LWS opsins, one SWS1 opsin, two SWS2 opsins, and three RH2 opsins [17]. Phylogenetic relationships suggest that these additional opsin duplication events occurred independently in the lineages leading to these two fishes [17], though this pattern may also reflect concerted evolution, resulting in homogenized gene sequences within species [18]. Though it is tempting to suggest that these increased opsin numbers reflect one of the whole genome duplication events thought to have occurred in the fish lineage [19], this does not appear to be the case; evidence from whole genome sequencing projects and the sequencing of opsins from genomic libraries suggests that the SWS2 and LWS opsins are consistently arranged in one tandem array, while the multiple RH2 opsins are arranged in another (zebrafish *D. rerio *[16]; medaka *O. latipes *[17], Lake Malawi cichlids [20], cavefish *Astyanax fasciatus *[21], and the pufferfish *Tetraodon nigroviridins *[19]). This pattern is consistent with shared opsin duplication events occurring early in these fishes' evolution, but through local duplication events, not genome duplications. In many cases, the duplicated opsins display numerous amino acid substitutions and substantial differences in spectral sensitivity (e.g., the RH2 opsins of zebrafish, whose spectral sensitivity maxima are located from 467 nm to 505 nm; [16]), which is indicative of functional divergence following the duplication events.

In this paper we describe our investigation of the LWS opsins of the guppy, *Poecilia reticulata*. The guppy has long served as a model system for researchers interested in natural and sexual selection, and is notable among fish in that it displays extremely high levels of variation for a number of traits, both behavioural and morphological [22,23]. Both male colour patterns [24] and female mate preferences for these colour patterns [25], for instance, vary dramatically among individuals within and among populations. Furthermore, microspectrophotometry (MSP) analysis of the guppy's photoreceptor cells revealed visual system variation; guppies appears to possess three photoreceptors maximally sensitive to different wavelengths of long-wavelength light, but the retina of individual fish may have different numbers and combinations of these three photoreceptors [26,27]. It was suggested that these three different photoreceptors reflect the presence of two spectrally-distinct LWS visual pigments, expressed either independently or in conjunction [27]. We characterized the LWS opsins of the guppy using degenerate primers to amplify LWS opsin fragments from a cDNA library constructed from a single individual. Surprisingly, our results indicate that guppy LWS opsins have experienced several recent rounds of duplication and divergence, and reveal the presence of at least six expressed LWS opsin sequences in a single individual.

## Results and Discussion

**Figure 1:**
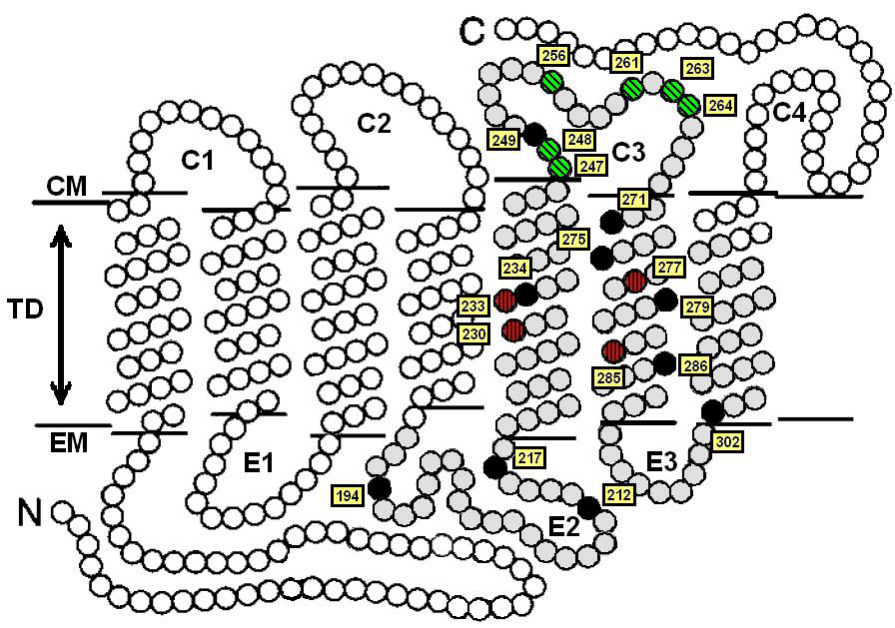
**Schematic diagram of an LWS opsin, with each circle representing an amino acid residue**. The portion of the guppy opsins isolated in this study is shaded grey. Sites found to be variable among the guppy opsins that were targeted in Franke et al.'s mutagenesis study of transducin activation capacity [48] are green with diagonal hatching. Those shown to be involved in spectral tuning [6, 45-47] are marked in red with vertical hatching. All other variable sites in the guppy opsins are shaded black. All amino acid numbering follows the human LWS opsins. The cytoplasmic (CM) and extracellular (EM) membranes, the transmembrane domain (TD), and the three extracellular (E1–E3) and four cytoplasmic (C1–C4) loops are indicated. Transmembrane boundaries were inferred by analogy to those of the bovine rhodopsin crystal structure [65].

We amplified and sequenced, from cDNA obtained from a single guppy individual, six LWS opsin sequences corresponding to a 390 bp fragment of the LWS opsin (designated as 'guppy LWS1–6'; Genbank accession numbers DQ865167–DQ865172). This fragment of the opsin corresponds to LWS exons 4 and 5, and codes for the portion spanning from the beginning of the second extracellular loop to the middle of the seventh transmembrane domain (Fig. 1). Variation within this portion of the gene is known to have a disproportionately large effect on visual pigment phenotype [6]. Most notably, this fragment contains transmembrane domains 5 and 6, which include many of the known LWS spectral tuning sites, including residues 277 and 285 that account for much of the difference between human red and green visual pigments [6]. This fragment also contains the third cytoplasmic loop, which is thought to strongly influence the interaction between the activated visual pigment and the downstream G-protein [3,4].

In order to eliminate errors due to enzyme misincorporations, template switching and other PCR artifacts, multiple clones from separate PCR reactions were sequenced, and an error rate of ~0.2% was allowed among the clones (see Methods for error rate calculations, and number of clones sequenced). One of the opsins, LWS5, which may be expressed at low levels, was only isolated once from cDNA, but several clones were isolated and sequenced from genomic DNA. The other five LWS opsin sequences obtained from cDNA were confirmed by designing primers specific to each LWS sequence, and performing amplifications from genomic DNA as well. These genomic amplification products were then sequenced to confirm they were the same as those isolated from cDNA.

**Figure 2:**
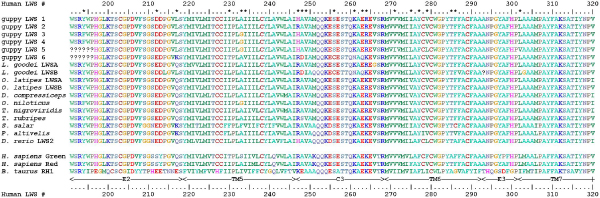
**Amino acid alignment of guppy LWS opsins with a sample of fish LWS opsins**. Human red and green LWS opsins, and bovine (RH1) rhodopsin, are also included for reference. Numbering corresponds to human LWS opsins. Structural elements (transmembrane domains (TM), and extracellular (E) and cytoplasmic (C) loops) are indicated beneath the alignment. Sites variable among guppy LWS opsins are marked with an asterisk. Genbank accession numbers for the LWS sequences used to create the alignment: *Lucania goodei *LWSA (AY296740), *Lucania goodei *LWSB (AY296741), *Oryzias latipes *LWSB (AB223052), *Oryzias latipes *LWSA (AB223051), *Dimidiochromis compressiceps *(AF247131), *Oreochromis niloticus *(AF247128), *Tetraodon nigroviridis *(AY598943), *Takifugu rubripes *(AY598942), *Salmo salar *(AY214131), *Plecoglossus altivelis *(AB107771), *Danio rerio *LWS2 (AB087804).

Among the six opsin sequences found, there were a total of 59 variable nucleotide sites and 20 variable amino acid sites (Fig. 2). Percentage sequence differences among the guppy LWS opsin sequences ranged from 1.04% to 14.3 % (distances computed with the method of Hasegawa et al. (HKY) [28]; Additional file 1). All guppy sequences had functionally critical residues known to be conserved among vertebrate opsins, such as the highly conserved lysine to which the chromophore is covalently bound via a Schiff-base linkage (Lys312; all numbering according to human LWS opsin), and a cysteine residue (Cys207) involved in a disulphide linkage with a residue in the third transmembrane helix, Cys126 [3,4,6]. These observations, along with the fact that the sequences were obtained from mRNA expressed in the head, suggest that the guppy LWS opsins are functional.

**Figure 3:**
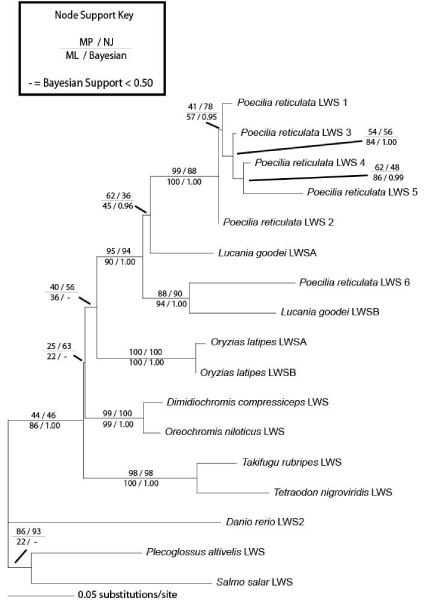
**Phylogeny showing the relationship between the LWS opsins of the guppy (*Poecilia reticulata*) and other teleost fishes**. This topology represents one of ten most parsimonious trees found (see Methods for details). Branch lengths were estimated for this topology using maximum likelihood methods under the HKY+G model. Bootstrap support values are indicated above each node for parsimony (upper left), neighbour-joining (upper right), maximum likelihood (lower left) and Bayesian analysis (lower right). Except for some of the basal nodes for which there was little support, particularly in the likelihood and Bayesian analyses, this tree was congruent among all the different types of analyses used.

**Figure 4:**
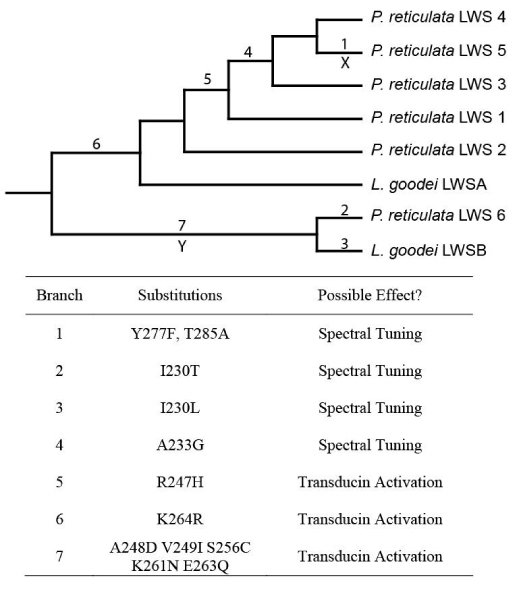
**Cladogram showing the relationships among guppy (*Poecilia reticulata*) and bluefin killifish (*Lucania goodei*) LWS opsins, with substitutions of likely functional importance mapped on the tree**. These substitutions were the result of most parsimonious ancestral reconstructions inferred using MacClade v4.0 [62]. The branch leading to guppy LWS5, marked with an X, and the branch leading to guppy LWS6 and *L. goodei *LWSB, marked with a Y, were analyzed in PAML using branch-specific and branch-site models.

We aligned the guppy LWS opsins with a sample of teleost fish LWS opsin sequences (Fig. 2), including the medaka (*Oryzias latipes*) [17], two cichids (*Dimidiochromis compressiceps *and *Oreochromis niloticus*) [20], two pufferfish (*Tetraodon nigroviridis *and *Takifugu rubripes*) [29], and a close relative of the guppy, the bluefin killifish (*Lucania goodei*) [30]. LWS opsins from the more distantly related ayu smelt (*Plecoglossus altivelis*) [31], Atlantic salmon (*Salmo salar*) [32], and zebrafish (*Danio rerio*) [16] were also included as outgroups. Figure 3 presents the results of phylogenetic analyses performed on this alignment. Though some nodes are weakly supported, the relationships among the ingroup acanthopterygian fish are consistent with current phylogenetic hypotheses for this group [33,34]. Interestingly, the LWS opsins of the guppy and the killifish, which is currently known to have at least two LWS opsins, display trans-specific evolution; that is, guppy LWS6 is most closely related to *L. goodei *LWSB while guppy LWS1–5 are most closely related to *L. goodei *LWSA (Figs. 3, 4). This pattern suggests that a LWS opsin duplication event (either involving the divergence of alleles within a locus, or the establishment of separate loci) pre-dated the guppy-killifish split, and that four more duplication events have occurred more recently within the guppy lineage. Whether or not all of the different guppy LWS opsin sequences represent products of true gene duplication events, or instead divergence among alleles within a single gene locus, is not currently known, but the results suggest that the guppy has at least three and possibly as many as six LWS opsin loci.

Observing this many distinct LWS opsin sequences in a single individual guppy is a highly surprising result. Not only is this number larger than the estimate derived from MSP analysis of guppy LWS photoreceptor cells [27], but it is, to our knowledge, greater than has been found for any other fish studied to date. Whole genome sequencing or screening and mapping of genomic libraries has confirmed single LWS opsins in two species of pufferfish [29], and two LWS opsins in both the zebrafish [16] and medaka [17], while screening and Southern blotting of a genomic library suggests the presence of two (or possibly three) LWS loci in the cavefish (*Astyanax fasciatus*) [21]. In humans, the LWS opsins are located tandemly along the X-chromosome; this tandem array expands and contracts as a result of non-homologous recombination events [6], and individuals can have from one to six X-linked LWS opsins per X chromosome [35,36]. The genomic organization of guppy LWS opsins is not currently known, but if guppy LWS opsins are arranged in a tandem array then it is possible that similar mechanisms may have generated the larger number of guppy LWS opsins.

Evolutionary patterns of selective constraint were investigated in our data set using codon-based likelihood phylogenetic models, as implemented in PAML [37,38]. Estimating the ratio of non-synonymous to synonymous rates (dN/dS or ω) in a single parameter model (M0) on the tree shown in Fig. 3 revealed a low overall dN/dS ratio of 0.08737 (Table 1). This is indicative of fairly strong purifying selection across sites, a result typical of most studies of proteins with highly conserved functional roles [39]. Models that allow ω to vary across sites were implemented in order to test for sites that may be under positive selection [40-42]; however, none of these tests were able to detect evidence of positive selection in our data set. Models incorporating site classes under positive selection were not found to be significantly better than neutral models in likelihood ratio tests, including M1a/M2a, and M7/M8 comparisons (Table 1). Only the M0/M3 comparison was significant, though this is not a strict test of positive selection. For the M3 model, the third site class was found to have a greatly elevated dN/dS ratio, though even in this case it remains below 1 (ω_2 _= 0.64).

Branch [43] and branch-sites [42,44] models were also implemented in order to test for changes in selective constraint along lineages leading to the more divergent sequences, LWS5 and LWS6 (Fig. 3). Both lineages were found to have a statistically significant, or nearly significant, increase in dN/dS ratio (over 5-fold in both cases; Table 1). These ratios were found to be even more elevated in branch-sites models (LWS5, ω_2 _= 16.5; LWS6, ω_2 _= 1.0), although the addition of this site class was not found to be statistically significant in either lineage (Table 1).

The significant changes in selective constraint in some of the LWS opsin lineages led us to examine in further detail sites that may be important in shifting opsin function in our data set. Several LWS opsin spectral tuning sites have been identified through comparative sequence analysis and site-directed mutagenesis studies [6,45-47]. Among the guppy LWS opsins identified in this study, variation exists at several sites known to be functionally important (Fig. 4; Table 2). Most notable are the differences at sites 277 and 285; guppy LWS5 possesses the same residues at those sites as the human green-LWS opsin (phenylalanine and alanine, respectively), while guppy LWS1–4,6 possess the same residues as the human red-LWS opsin (tyrosine and phenylalanine). These two substitutions are known, through mutagenesis, to cause a relatively large spectral shift of approximately 15–25 nm, and are the primary substitutions responsible for the functional difference between human red and green LWS opsins [6,45-47]. This strongly suggests that the LWS5 may shifted toward shorter wavelengths (*i.e.*, more toward the green) relative to the other guppy LWS opsins isolated in this study. This shift in function may be the reason for the significantly elevated dN/dS ratio found in codon-based models allowing for an additional ω parameter along this branch (Table 1). Moreover, branch-sites models identified both residues 277 and 285 as possible targets of positive selection, though inclusion of this class of positively selected sites was not found to be statistically significant (Table 1).

Sites 230 and 233, minor spectral tuning sites, are also variable in some of the sequences in our data set (Fig. 4, Table 2). At site 230, guppy LWS6 possesses a threonine, while the rest possess an isoleucine; this mutation was found to alter spectral sensitivity by a few nm in human LWS opsins [45]. At site 233, guppy LWS3–5 possess a glycine, while the rest possess an alanine; mutagenesis studies have shown that substituting a serine for an alanine at this site does have a small effect on spectral sensitivity in human LWS opsin [45]. The effects of such substitutions on spectral sensitivity are small enough that they are often undetected by methods such as MSP, which could explain why MSP studies of guppy photoreceptor cells suggested a relatively low number of visual pigments (2) sensitive to long-wavelength light [26,27] relative to the higher number of LWS opsin sequences (6) found in this study. There are also additional substitutions observed among guppy LWS opsin sequences that have not yet been explored by mutagenesis methods which may influence spectral sensitivity as well (for example, site 234, which is immediately adjacent to the minor spectral tuning site 233). Such mutations will require experimental evaluation through site-directed mutagenesis and spectrophotometric analysis of expressed visual pigments [45].

Variation also exists among guppy LWS opsin sequences at sites that likely influence other, non-spectral properties of the visual pigment (Fig. 4, Table 2). The most notable differences occur within the third cytoplasmic loop, a portion of the visual pigment known to be critically important for binding and activating transducin following the absorption of a photon of light [48,49]. Guppy LWS6 differs from the rest at seven AA sites within this loop. The effect of several of these sites on transducin activation rates was experimentally tested in mutated bovine rhodopsin expressed *in vitro *[48] (Table 2). The effect of most substitutions was to reduce transducin activation in the mutants relative to wildtype bovine rhodopsin. The physiochemical properties of residues at sites 247, 248, and 264, guppy LWS6 are more similar to the wildtype bovine rhodopsin, suggesting that guppy LWS6 may have an enhanced transducin activating ability as compared with other guppy LWS opsins. However, the residues possessed by guppy LWS6 at sites 256, 261, and 263 appear to be unique among vertebrate opsins, with all other vertebrate opsins being either invariant or possessing residues with fairly different physiochemical properties.

The resemblance of the residues in guppy LWS6 at key sites with rhodopsin, combined with the known importance of the third cytoplasmic loop in G-protein binding and activation [49,50], raise the interesting possibility that the guppy LWS6 opsin may have divergent function with regards to transducin activation relative to other LWS opsins. Many of these same substitutions in the third cytoplasmic loop are present in *L. goodei *LWSB, the killifish opsin most closely related to guppy LWS6, including the unique substitutions at site 256, 261, and 263 (Fig. 3, Table 2), suggesting that variation in transducin activating capacity is being selectively maintained. This is consistent with our codon-based analyses using branch-specific models, which revealed a nearly statistically significant, 5.4-fold increase in ω along the branch leading to guppy LWS6 and *L. goodei *LWSB (Fig. 4), even if the branch-site analysis of this lineage did not provide any evidence of positive selection (Table 1). Interestingly, Bayes Empirical Bayes [42] analysis did identify five residues (including sites with known influence on transducin activation [48]), all within the third cytoplasmic loop (residues 248, 249, 256, 261 and 263).

The evolutionary mechanisms that have promoted and maintained this diversity of LWS opsins in the guppy remain to be investigated. Guppies are known to have highly variable colour patterns and mate preferences [22]. The chroma of orange patches on male guppies varies among individuals, and it has been suggested that these differences may reflect differences in male foraging ability or parasite load [22]. By having multiple LWS opsins with slight variation in spectral sensitivity, female guppies may be able to better discriminate among differently coloured males [51,52] or the brightly-coloured fruits that occasionally fall into the guppy's streams [53]. Also, it has been observed in the cichlid *Metriaclima zebra *that a region of the cichlid genome that influences colour pattern is closely linked to the SWS2-LWS opsin tandem array [54]; selection acting to maintain colour pattern variation could potentially lead to the maintenance of variation in physically linked opsin genes. Both directional [22,25] and balancing [55,56] selection have been shown to operate on variation in male colour patterns in the guppy, and could therefore function to influence visual variation if a similar linkage exists in the guppy. In future work, we plan to investigate the relationship between behavioural ecology and opsin molecular biology in the guppy by studying the influence of opsin genes on individual behaviour and fitness.

## Conclusion

Our results demonstrated the presence of at least six expressed LWS opsin sequences within a single guppy individual. This number is substantially higher than the number found in any other fish to date, and higher than the minimum number previously estimated for the guppy by MSP. Phylogenetic analysis reveals that these guppy LWS opsins fall into two groups, one group of relatively closely related sequences, and another that appears to have descended from a duplication event pre-dating the guppy-killifish split. Many of the amino acid substitutions that distinguish these guppy LWS opsins appear at sites thought to be functionally important, either for spectral tuning or transducin activation. This is consistent with codon-based analyses, which indicate changes in selective constraint along two of the more divergent lineages of guppy LWS opsins.

## Methods

Total RNA was extracted from the head of a single male guppy sampled from a tributary of the Paria River, Trinidad. The fish was anesthetized in MS-222 solution, its head dissected and ground on dry ice, and the RNA isolated using an RNeasy Mini Kit (Qiagen). Single stranded mRNA was converted to double stranded cDNA via reverse transcriptase (SMART cDNA Library construction kit; BD Bioscience). Both primer extension and low-density PCR strategies were employed (involving 9 to 30 amplification cycles per reaction) to generate double stranded cDNA. Internal fragments of the guppy's LWS opsin coding sequence were amplified (43 amplification cycles per reaction) using FastStart Taq polymerase (Roche) and degenerate primers designed from conserved regions of published vertebrate M/LWS opsins (Forward primers VertRG168F 5'-TGC GCT CCT CCN ATH TTY GG and VertRG173F 5'-TTT GGA TGG TCN CGN TAY TGG CC, and Reverse primer VertRG313R 5'-GCG GAA CTG TCG ATT CAT RAA NAC RTA DAT). Given the number of amplification cycles and the error rates of the enzymes, this procedure is expected to yield an error rate of approximately 10^-3^, or about 0.53 nucleotide misincorporations per 390 bp fragment amplified. Amplified fragments were then cloned into plasmid vectors using TA cloning kits (Invitrogen), purified via spin columns (Qiagen and Eppendorf), and sequenced with modified M13 vector primers on an ABI Prism 3100 sequencer. For each LWS opsin isolated, multiple clones from separate PCR reactions were sequenced in both directions to control for enzyme misincorporations, template switching and sequencing artifacts (LWS1, 29 clones; LWS2, 12 clones; LWS3, 15 clones; LWS4, 6 clones; LWS5, 1 clone; LWS6, 9 clones). In order to confirm the existence of the different LWS opsin sequences found in cDNA, portions of the opsin that distinguished the guppy LWS sequences from each other were also amplified from genomic DNA using specific primers, and sequenced. For the LWS5 opsin, although only one cDNA clone was isolated, multiple clones (7) were sequenced from genomic DNA amplifications.

Vertebrate LWS opsin sequence alignment was carried out via ClustalW [57]. Amino acid sequences were aligned, and from this the nucleotide alignment was inferred. Preliminary rounds of phylogenetic analysis (Neighbour-joining method), carried out separately on the upstream and downstream halves, revealed a small number of clones as likely instances of recombination among clones during amplification; these clones were excluded from further analysis. The low amount of statistical power afforded by the small size of the dataset precluded the use of more refined methods for detecting instances of template switching. Several different methods of phylogenetic analyses were performed on the alignment shown in Figure 2, with smelt (*P. altivelis*), salmon (*S. salar*), and zebrafish (*D. rerio *LWS2) LWS opsins used as outgroups. Hierarchical likelihood ratio tests, implemented in Modeltest 3.6 [58], identified the HKY+G model as the best fitting model. Neighbour-joining, maximum parsimony, and maximum likelihood analyses were carried out in PAUP* 4.0b10 [59]. Parsimony heuristic searches were implemented with 1000 random addition replicates. Node support was assessed using bootstrap analysis [60], as implemented in PAUP* 4.0b10 [59], for the neighbor-joining (1000 replicates, HKY+G model), parsimony (1000 replicates), and maximum likelihood analyses (100 replicates, HKY+G model). Bayesian analyses were carried out in Mr. Bayes 3.1.1 [61]. Four simultaneous chains were run for 10^7 ^generations, with tree sampling every 100 generations (for a total of 100000 trees). The first 5000 trees were considered 'burn-in' and discarded. Most parsimonious ancestral reconstructions, and mapping of amino acid substitutions onto the phylogeny were carried out using MacClade v4.0 [62].

Evolutionary patterns of selective constraint were assessed using codon-based likelihood analyses as implemented in PAML v3.15 [38], using the aligned nucleotide dataset and the tree topology shown in Fig. 3. PAML uses a maximum likelihood framework to estimate dN/dS (ω) for the entire dataset and tree (M0 model), for particular lineages (branch models), for particular codons (sites models), or for particular codons within particular lineages (branch-site models) (reviewed in [37]; also see [40-44] for specificities on the various models employed). The ω value is thought to reflect the degree of evolutionary constraint, or selection pressure operating on the site class and/or lineage of interest, with 0 < ω < 1 indicating purifying selection, ω = 1 neutral evolution, and ω > 1 positive selection [63]. Nested models are compared via likelihood ratio tests, with the test statistic following a χ^2 ^distribution with degrees of freedom equal to the difference in the number of parameters between the two models [60,64]. Likelihood ratio tests were carried out using the program chi2, which is bundled with the PAML software package [37]. Bayes Empirical Bayes analysis, as implemented in PAML, was used to identify particular residues within the dataset as likely targets of positive selection [42].

## List of abbreviations

LWS = long-wavelength sensitive, SWS1 = type 1 short-wavelength sensitive, SWS2 = type 2 short-wavelength sensitive, RH1 = rod, RH2 = rod-like (or medium-wavelength sensitive), MSP = microspectrophotometry

## Authors' contributions

CJW performed the molecular laboratory work, compiled the data, performed analyses, and drafted the manuscript. BSWC conceived of and guided all aspects of the study, performed some of the analyses and the lab work, and helped with the drafting of the manuscript.

## Supplementary Material

Additional file 1 HKY corrected (beneath diagonal) and uncorrected p-distance (above diagonal) data matrix. Click here for file

## Figures and Tables

**Table 1:** PAML results: log likelihoods, parameter estimates, Bayes Empirical Bayes identified sites, and likelihood ratio tests.

Selected Branch	Model	n.p.	*ln*L	Parameter Estimates	Bayes Empirical Bayes Identified Sites *	Likelihood Ratio Test P-value
N/A	M0 (one-ratio)	33	-2127.37	ω = 0.08737	none	N/A
	**Site-specific models**					
	M1a (neutral)	34	-2097.31	*p*_0 _= 0.91742, ω_0 _= 0.05054	N/A	N/A
	M2a (selection)	36	-2097.31	*p*_0 _= 0.91768, ω_0 _= 0.05052, *p*_2 _= 0.04090, ω_2 _= 0.99980	275	P = 1 (vs. m1a)
	M3 (discrete, *K *= 3)	37	-2088.38	*p*_0 _= 0.53422, *p*_1 _= 0.38154, ω_0 _= 0.00000, ω_1 _= 0.12511, ω_2 _= 0.63618	none	P < 0.000001 (vs. m0)
	M7 (β)	34	-2089.48	p = 0.21896, q = 1.82556	N/A	N/A
	M8 (β & ω)	36	-2088.91	p = 0.24125, q = 2.23372, *p*_0 _= 0.99249, ω = 1.66242	275	P = 0.568523 (vs. m7)
	**Branch-specific models**					
X 'green'	two-ratio	34	-2123.10	ω_0 _= 0.0806, ω_1 _= 0.4259	N/A	P = 0.003463 (vs. m0)
Y 'transducin'	two-ratio	34	-2125.68	ω_0 _= 0.0832, ω_1 _= 0.4523	N/A	P = 0.066388 (vs. m0)
	**Branch-site (BS) models**					
X 'green'	BS model A (null)	35	-2094.55	*p*_0 _= 0.73148, ω_0 _= 0.04521, *p*_1 _= 0.06817	N/A	N/A
	BS model A (alternative)	36	-2093.93	*p*_0 _= 0.86007, ω_0 _= 0.04576, *p*_1 _= 0.06817, ω_2 _= 16.53309	271, 275, 277, 279, 285, 286, 302	P = 0.266644 (vs. BS model A [null])
Y 'transducin'	BS model A (null)	35	-2093.10	*p*_0 _= 0.31761, ω_0 _= 0.04432, *p*_1 _= 0.03013	N/A	N/A
	BS model A (alternative)	36	-2093.10	*p*_0 _= 0.32723, ω_0 _= 0.04442, *p*_1 _= 0.03012, ω_2 _= 1.00000	**248, 249, 256, 261, 263**	P = 1 (vs. BS model A [null])

n.p. = number of parameters (e.g., proportions, ω values, branch lengths).

*ln*L = log likelihood value for model given the tree, dataset, and parameter estimates.

* = sites in bold received posterior probability support > 0.9.

Test statistic compared to ac2 distribution with d.o.f. = n.p.alternative - n.p.null

See Fig. 4

**Table 2:** Substitutions observed among guppy LWS opsins, and comparable mutants made in bovine RH1 and human LWS.

Observed Substitutions Among Guppy Opsins^1^	Comparable Mutants Made in Bovine RH1^2 ^[48]	Transducin Activity Relative to Wildtype RH1 [48]
S256C	S256A	60%
K261N	K261L	98%
R264K	K264L	72%
H247R, A248D	K247T, E248Q	87%
K261N, R264K	K261L, K264L	61%
E263Q, R264K	E263Q, K264L	110%

Observed Substitutions Among Guppy Opsins^1^	Comparable Mutants Made in Human LWS^2 ^[45, 46]	Effect on LWS Spectral Tuning LWS [45, 46]

I230T	I230T	2–3 nm
A233G	A233S	2–3 nm
Y277F	Y277F	6–10 nm
T285A	T285A	10–16 nm
Y277F, T285A	Y277F, T285A	18–27 nm

^1 ^Substitutions observed among guppy LWS opsins; refer to text and Fig. 4 for more details

^2 ^Mutants created in bovine RH1 and human LWS by site-directed mutagenesis
