# Supplementary material for: Long-wavelength sensitive visual pigments of the guppy (Poecilia reticulata): six opsins expressed in a single individual
Source: BMC Evol Biol. 2007 Feb 8;7(Suppl 1):S11. doi: 10.1186/1471-2148-7-S1-S11 (PMC1796605; doi:10.1186/1471-2148-7-S1-S11)
Supplement: Additional file 1 — HKY corrected (beneath diagonal) and uncorrected p-distance (above diagonal) data matrix. [file 1471-2148-7-S1-S11-S1.doc]

Supplementary Table 1 – HKY corrected (beneath diagonal) and uncorrected p-distance (above diagonal) data matrix.

|  | **Name** | **1** | **2** | **3** | **4** | **5** | **6** | **7** | **8** | **9** | **10** | **11** | **12** | **13** | **14** | **15** | **16** | **17** |
| --- | --- | --- | --- | --- | --- | --- | --- | --- | --- | --- | --- | --- | --- | --- | --- | --- | --- | --- |
| **1** | **guppy LWS 1** | - | 0.0103 | 0.018 | 0.0282 | 0.0565 | 0.1255 | 0.0872 | 0.1206 | 0.118 | 0.1103 | 0.1744 | 0.1667 | 0.1256 | 0.1333 | 0.1795 | 0.1436 | 0.1539 |
| **2** | **guppy LWS 2** | 0.0104 | - | 0.0128 | 0.0231 | 0.0565 | 0.1202 | 0.0821 | 0.1154 | 0.1128 | 0.1051 | 0.1795 | 0.1615 | 0.1154 | 0.1231 | 0.1718 | 0.1359 | 0.1462 |
| **3** | **guppy LWS 3** | 0.0182 | 0.0129 | - | 0.0154 | 0.0511 | 0.1256 | 0.0872 | 0.1257 | 0.1205 | 0.1128 | 0.1846 | 0.1718 | 0.1256 | 0.1231 | 0.1795 | 0.1462 | 0.1564 |
| **4** | **guppy LWS 4** | 0.0289 | 0.0235 | 0.0156 | - | 0.0456 | 0.1201 | 0.0974 | 0.1283 | 0.1282 | 0.1205 | 0.1846 | 0.1718 | 0.1308 | 0.1282 | 0.1846 | 0.1462 | 0.1564 |
| **5** | **guppy LWS 5** | 0.059 | 0.0591 | 0.0532 | 0.0472 | - | 0.129 | 0.1129 | 0.1478 | 0.1505 | 0.1425 | 0.193 | 0.1985 | 0.1582 | 0.1585 | 0.2148 | 0.1666 | 0.1801 |
| **6** | **guppy LWS 6** | 0.1394 | 0.1327 | 0.1394 | 0.1325 | 0.1427 | - | 0.1338 | 0.1174 | 0.1739 | 0.1713 | 0.211 | 0.1949 | 0.147 | 0.1525 | 0.2057 | 0.1793 | 0.2006 |
| **7** | ***L. goodei* LWSA** | 0.0939 | 0.0879 | 0.0936 | 0.1057 | 0.1238 | 0.1504 | - | 0.1206 | 0.1205 | 0.1128 | 0.1949 | 0.1641 | 0.1154 | 0.1231 | 0.1923 | 0.1385 | 0.1513 |
| **8** | ***L. goodei* LWSB** | 0.1323 | 0.126 | 0.1383 | 0.1415 | 0.1657 | 0.1291 | 0.1321 | - | 0.1565 | 0.1539 | 0.1899 | 0.1719 | 0.1334 | 0.1411 | 0.1976 | 0.1796 | 0.1872 |
| **9** | ***O. latipes* LWSA** | 0.1303 | 0.1239 | 0.1331 | 0.1428 | 0.1703 | 0.2015 | 0.1329 | 0.1764 | - | 0.0077 | 0.1744 | 0.159 | 0.1103 | 0.1051 | 0.1897 | 0.1256 | 0.1487 |
| **10** | ***O. latipes* LWSB** | 0.1208 | 0.1145 | 0.1235 | 0.1331 | 0.1597 | 0.198 | 0.1234 | 0.173 | 0.0078 | - | 0.1769 | 0.1564 | 0.1077 | 0.1077 | 0.1872 | 0.1231 | 0.1462 |
| **11** | ***D. rerio* LWS2** | 0.1988 | 0.2056 | 0.2123 | 0.2123 | 0.2243 | 0.2488 | 0.2269 | 0.2192 | 0.1989 | 0.2024 | - | 0.1539 | 0.1744 | 0.1769 | 0.1949 | 0.1821 | 0.1872 |
| **12** | ***P. altivelis*** | 0.189 | 0.1823 | 0.1956 | 0.1956 | 0.2318 | 0.2271 | 0.1858 | 0.1956 | 0.179 | 0.1757 | 0.1732 | - | 0.1436 | 0.1436 | 0.1539 | 0.1692 | 0.1718 |
| **13** | ***D. compressiceps*** | 0.1401 | 0.1271 | 0.1396 | 0.1461 | 0.1808 | 0.1662 | 0.1273 | 0.148 | 0.1204 | 0.1173 | 0.199 | 0.1596 | - | 0.0256 | 0.1615 | 0.1385 | 0.1308 |
| **14** | ***O. niloticus*** | 0.1503 | 0.1371 | 0.1376 | 0.1442 | 0.1824 | 0.1742 | 0.1373 | 0.1576 | 0.1143 | 0.1174 | 0.2023 | 0.1595 | 0.0261 | - | 0.1564 | 0.1333 | 0.1359 |
| **15** | ***S. salar*** | 0.2093 | 0.1984 | 0.2086 | 0.2159 | 0.2581 | 0.2471 | 0.2293 | 0.2317 | 0.2233 | 0.2196 | 0.2273 | 0.1732 | 0.185 | 0.1778 | - | 0.1769 | 0.1821 |
| **16** | ***T. rubripes*** | 0.1617 | 0.1517 | 0.1646 | 0.1646 | 0.1908 | 0.2092 | 0.1553 | 0.207 | 0.1384 | 0.1352 | 0.2088 | 0.192 | 0.155 | 0.1487 | 0.2043 | - | 0.0718 |
| **17** | ***T. nigroviridis*** | 0.1751 | 0.1649 | 0.178 | 0.178 | 0.2101 | 0.2403 | 0.1726 | 0.2181 | 0.1677 | 0.1644 | 0.2162 | 0.1957 | 0.1454 | 0.1518 | 0.2108 | 0.0761 | - |
